# Supplementary figures and images for: Neurophysiological evidence for crossmodal (face-name) person-identity representation in the human left ventral temporal cortex
Source: PLoS Biol. 2020 Apr 3;18(4):e3000659. doi: 10.1371/journal.pbio.3000659 (PMC7159237; doi:10.1371/journal.pbio.3000659)

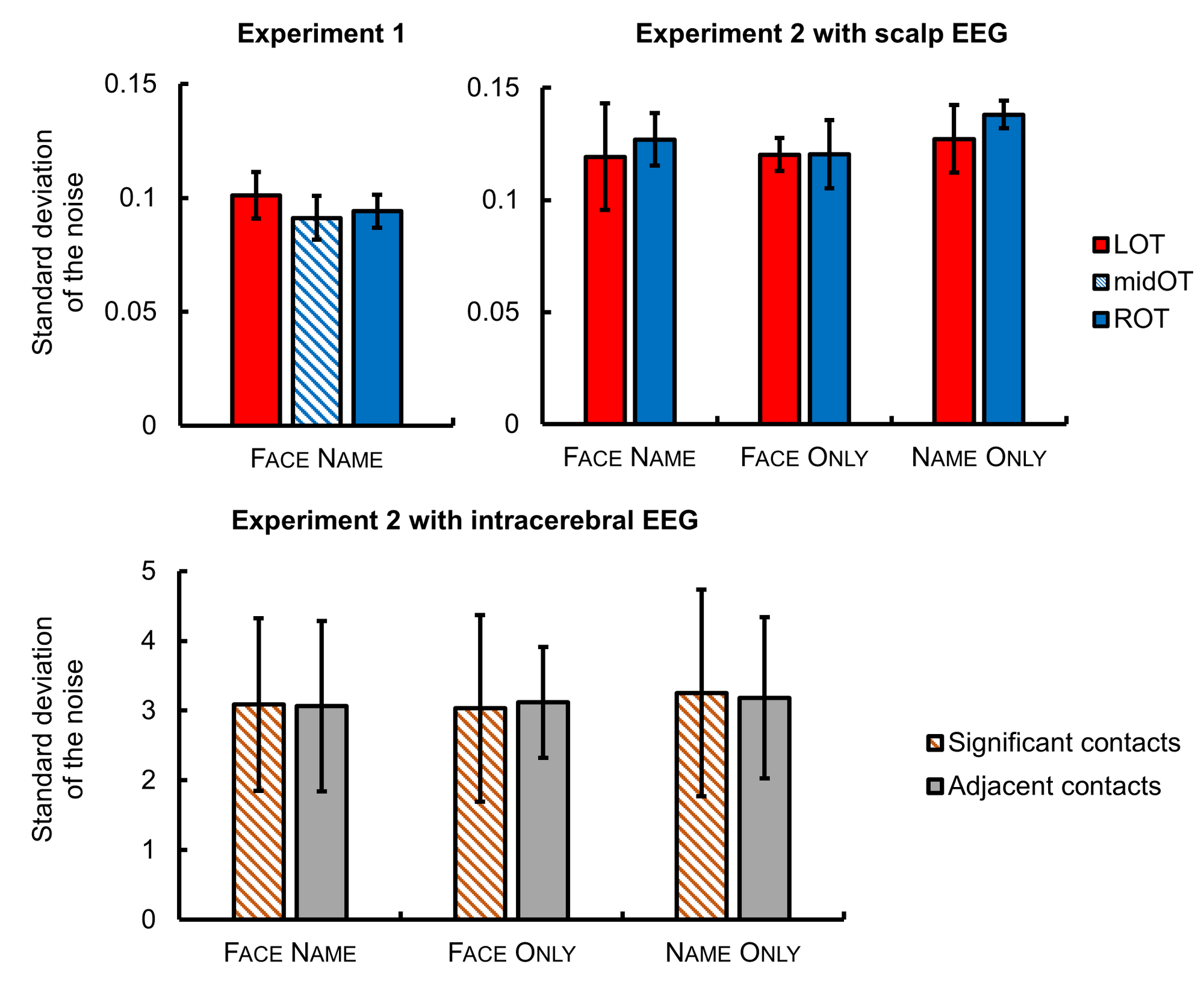

Supplement: S1 Fig — In Experiment 1, the mean standard deviation of the noise around the identity-oddball frequency is displayed for the left, middle, and right ROIs (3 electrodes in each). In Experiment 2 with scalp EEG, the mean standard deviation of the noise in the left and right ROIs (5 electrodes each) is shown for the 3 conditions: Face Name, Face Only, and Name Only. In Experiment 2 with intracerebral EEG, the mean standard deviation of the noise around the identity-oddball bin is shown for all significant contacts in the ATL and PTL (N = 15) and for all their nonsignificant adjacent contacts (N = 12) across all the 6 participants showing a significant response in at least one condition, either in the ATL or PTL. Error bars indicate standard deviation of the mean. Data underlying this figure are deposited on a Dryad repository: https://doi.org/10.5061/dryad.m8t391m. ATL, anterior temporal lobe; EEG, electroencephalography; LOT, left occipito-temporal; mid OT, middle occipito-temporal; PTL, posterior temporal lobe; ROI, region of interest; ROT, right occipito-temporal; SEEG, stereo electroencephalography. (TIF) [file pbio.3000659.s001.tif]

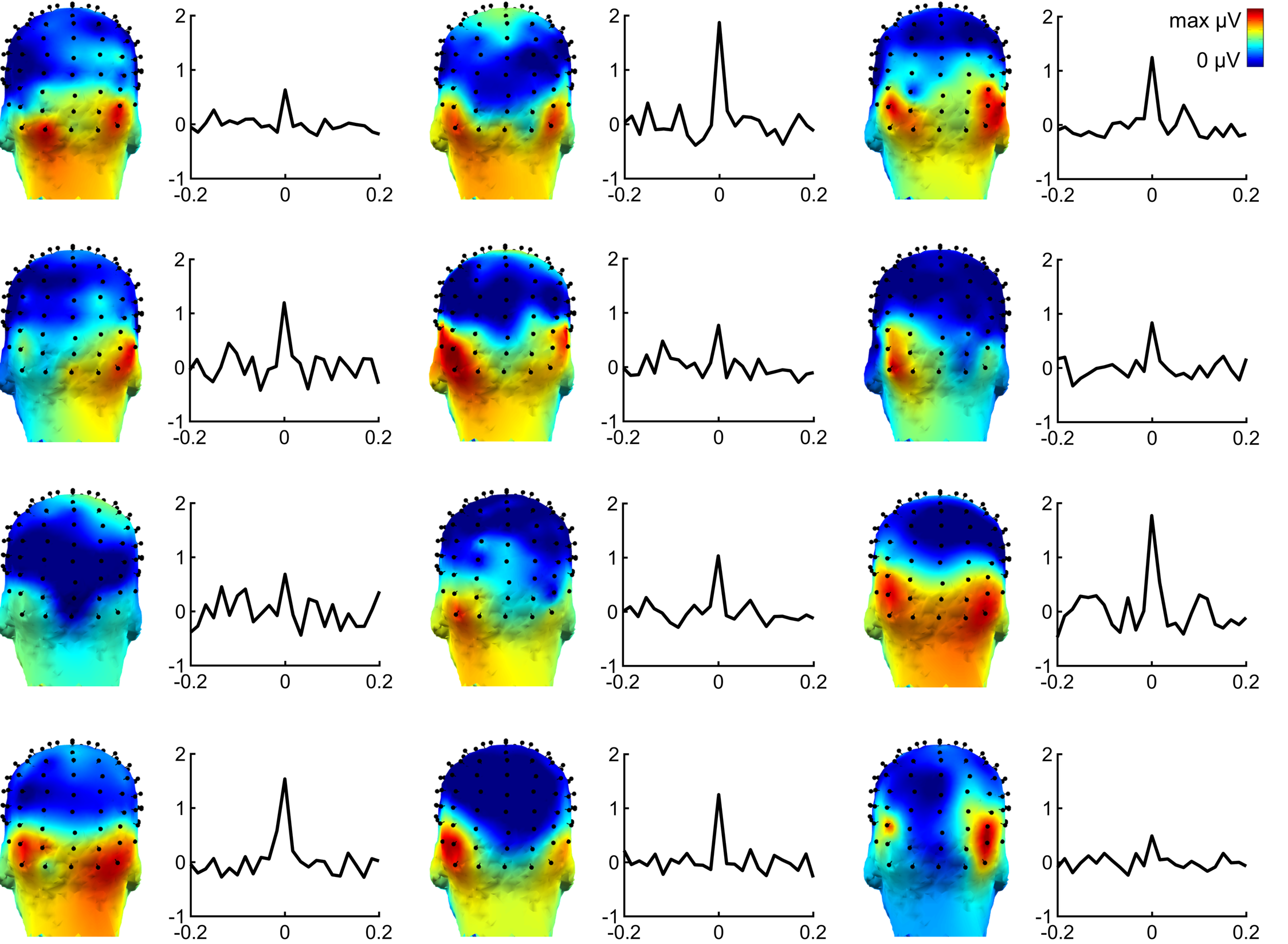

Supplement: S2 Fig — Individual participant data for the 12 participants of Experiment 1 are shown at the frequency of person identity change (sum of 6 harmonics), independently of base identity. Each topography and the waveform to its right correspond to the data of one participant. Head plots are scaled from 0 μV (dark blue) to the voltage of the maximal channel (red), separately for each participant. Waveforms show the average of baseline-corrected chunked frequency spectrum of the 6 channels in bilateral occipito-temporal ROIs (P10, PO10, and PO12 for the LOT, and P9, PO9, and PO11 for the ROT), centered on the sum of harmonics, with an x axis of relative frequency in Hz and a y axis of amplitude (μV). All participants showed a significant response (p < 0.01) at the frequency of person identity change in the bilateral ROIs. Data underlying this figure are deposited on a Dryad repository: https://doi.org/10.5061/dryad.m8t391m. EEG, electroencephalography; LOT, left occipito-temporal; ROI, region of interest; ROT, right occipito-temporal. (TIF) [file pbio.3000659.s002.tif]

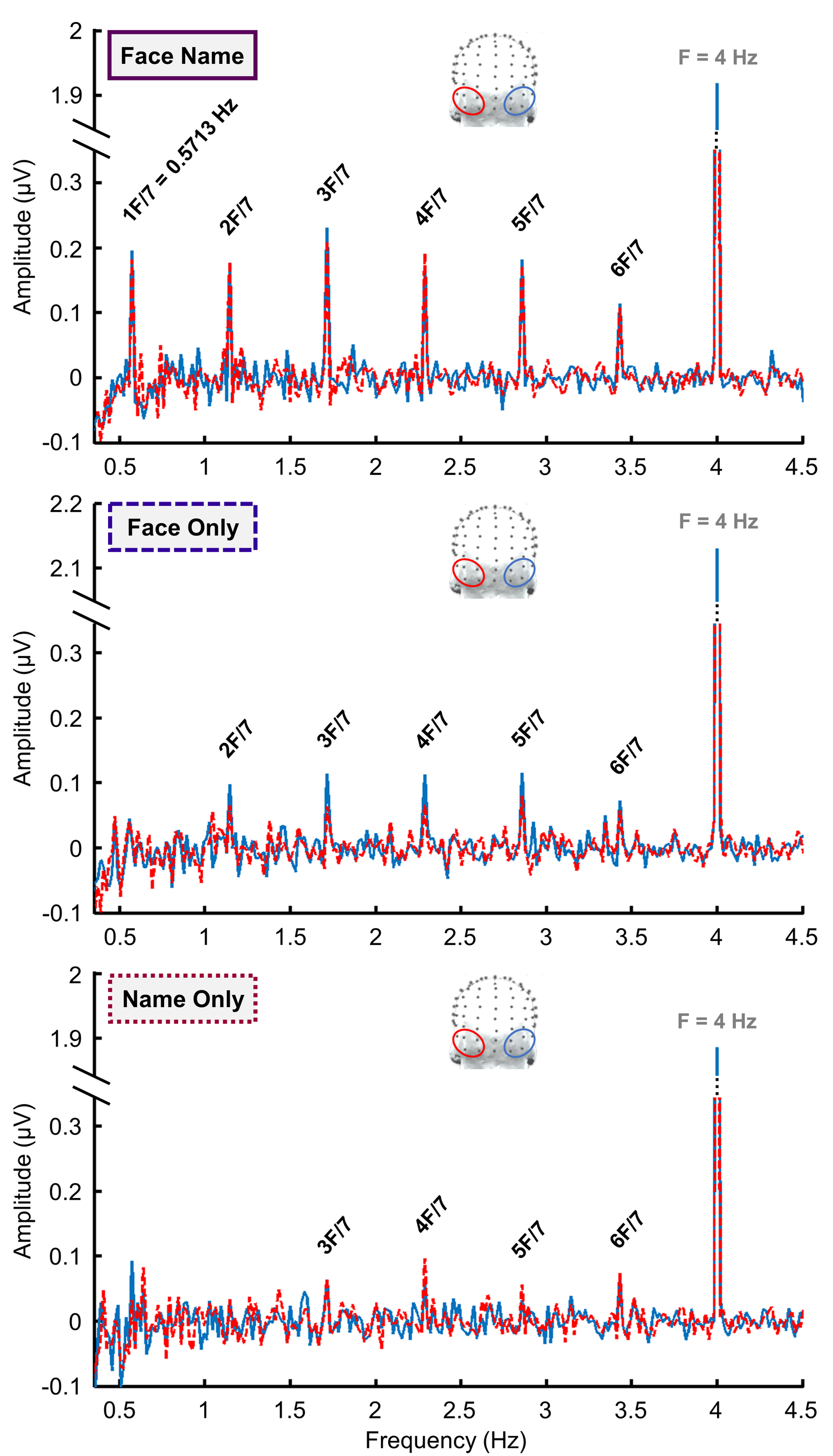

Supplement: S3 Fig — Baseline-corrected FFT spectra (in μV) of the responses in the 3 conditions of Experiment 2 are shown at the group level (n = 20 participants). The red and blue lines represent the average of the 5 electrodes in the left and right ROIs, respectively; the location of the ROI is indicated by the topographical head on the top of each spectrum. Black labels on the FFT spectrum signal the significant oddball frequencies; the light gray label indicates the base frequency. Data underlying this figure are deposited on a Dryad repository: https://doi.org/10.5061/dryad.m8t391m. EEG, electroencephalography; FFT, fast Fourier transform; ROI, region of interest. (TIF) [file pbio.3000659.s003.tif]

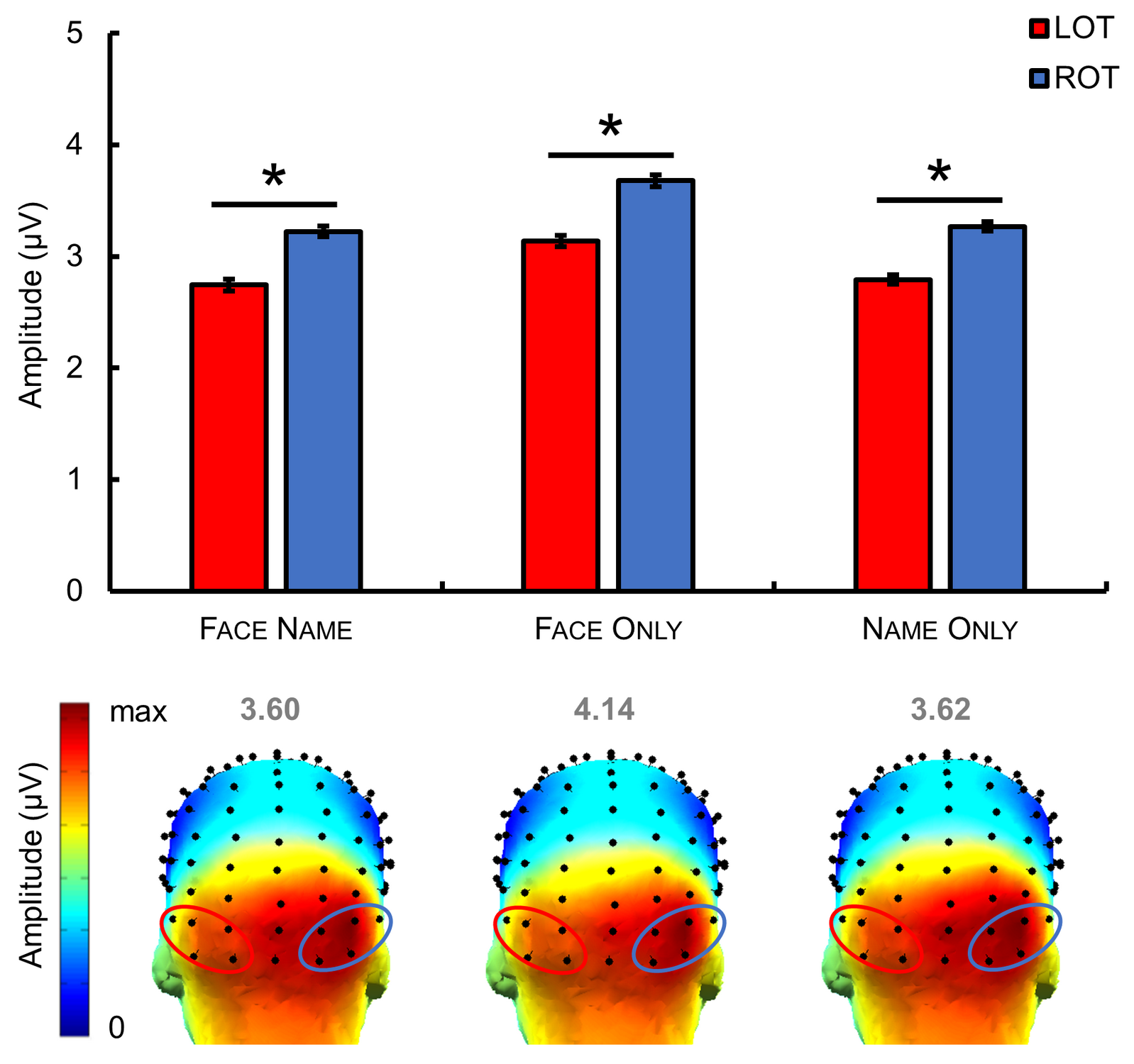

Supplement: S4 Fig — Base frequency responses (sum of the first 9 base harmonics) are shown for each of the 3 conditions: Face Name, Face Only, and Name Only. Above, mean group-level base responses in baseline-corrected amplitude (μV) at the two lateral ROIs regardless of identity. Error bars indicate standard error from the mean, reflecting variability across participants. Asterisks indicate significant differences at p < 0.05. Below, topography of the response at the base frequency for each condition. Red and blue ovals show the electrodes included in the left and right ROIs, respectively. The color scale maximum is shown in light gray above each map and corresponds to the maximal baseline-corrected amplitude (in μV) in each condition. Data underlying this figure are deposited on a Dryad repository: https://doi.org/10.5061/dryad.m8t391m. EEG, electroencephalography; LOT, left occipito-temporal; ROI, region of interest; ROT, right occipito-temporal. (TIF) [file pbio.3000659.s004.tif]

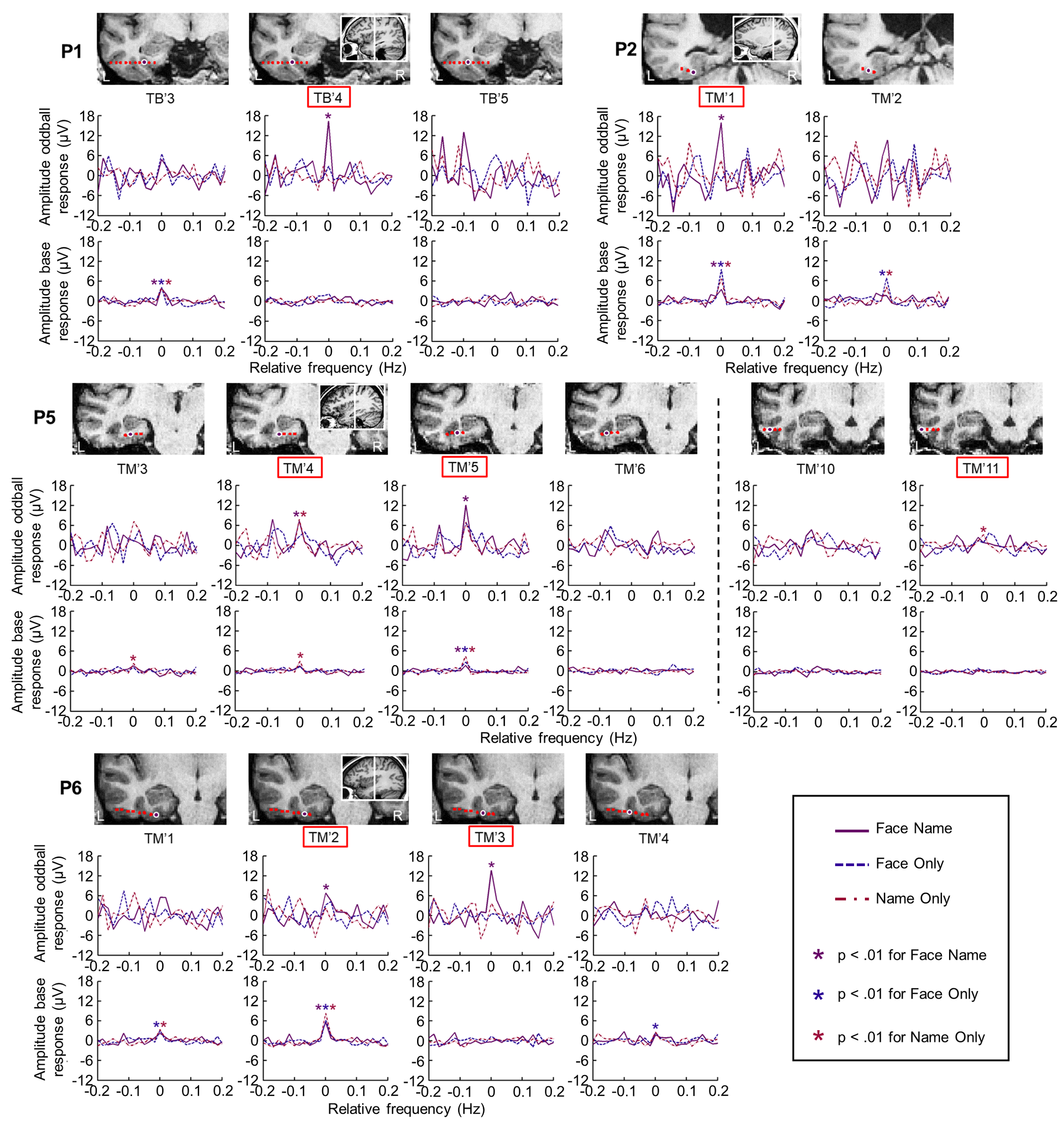

Supplement: S5 Fig — Anatomical location and electrophysiological responses of the 7 significant contacts in the ATL in 4 participants (P1, P2, P5, P6) and of their adjacent contacts. The significant contacts are highlighted in red. Significant base responses were determined in the same way as significant identity-oddball responses by (1) epoching the EEG frequency spectrum into segments centered on the first 6 base harmonics (i.e., 4 Hz, 8 Hz, etc.); (2) summing the amplitude values of these 6 frequency spectra segments; and (3) transforming it into a Z-score (difference between the amplitude at the base frequency bin and the mean amplitude of the 22 surrounding bins, divided by the standard deviation of amplitudes in the corresponding 22 surrounding bins). Data underlying this figure are deposited on a Dryad repository: https://doi.org/10.5061/dryad.m8t391m. ATL, anterior temporal lobe; EEG, electroencephalography; SEEG, stereo electroencephalography (TIF) [file pbio.3000659.s005.tif]

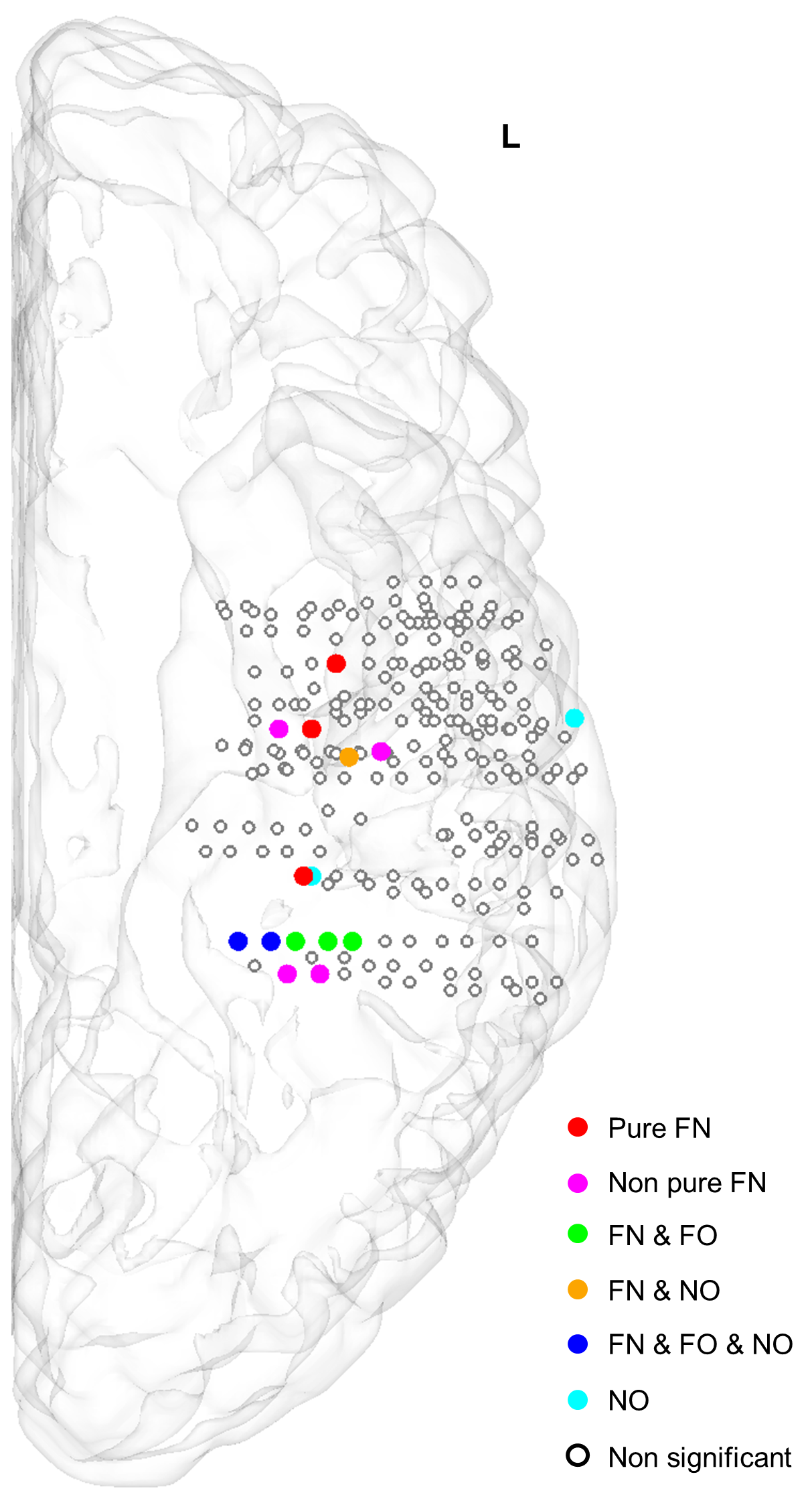

Supplement: S6 Fig — Map of all 237 VOTC recording contacts implanted in the gray matter of the left ATL and PTL across the 7 participants displayed in the Talairach space using a transparent reconstructed cortical surface of the Colin27 brain (left hemisphere, ventral view). Each circle represents a single recording contact. Color-filled circles correspond to significant contacts in at least one condition. Significant contacts are color-coded according to the condition(s) for which we recorded significant responses at the oddball-identity frequency (p < 0.001). “Pure FN” contacts are contacts on which the response was significant in the Face Name condition but not in the Face Only and Name Only conditions, and the response in the Face Name condition was larger (p < 0.05) than the response in the sum of the two control conditions. “Non pure” FN contacts are contacts on which the response was significant in the Face Name condition but not in the Face Only and Name Only conditions, but the response in the Face Name condition was not significantly larger than the sum of the two control conditions. Note that the more posterior “Pure” FN contact is still located in the ATL (i.e., anterior to the posterior tip of the hippocampus in the individual anatomy). Data underlying this figure are deposited on a Dryad repository: https://doi.org/10.5061/dryad.m8t391m. ATL, anterior temporal lobe; FN, Face Name; FO, Face Only; NO, Name Only; PTL, posterior temporal lobe; VOTC, ventral occipito-temporal cortex. (TIF) [file pbio.3000659.s006.tif]

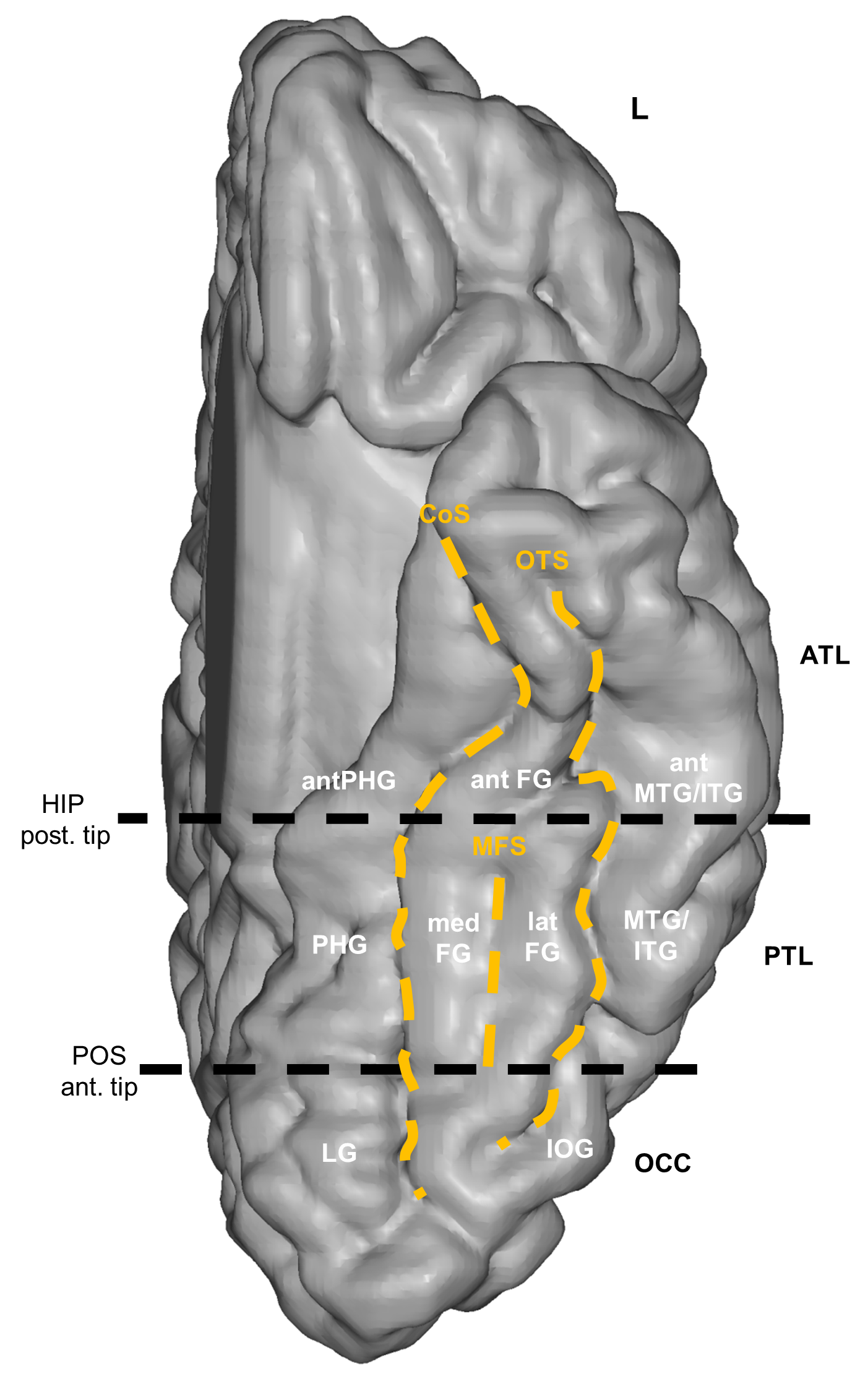

Supplement: S7 Fig — Anatomical regions were defined in each individual hemisphere according to major anatomical landmarks [95]. The ventral temporal sulci (collateral sulcus, occipito-temporal sulcus, and midfusiform sulcus) serve as medial/lateral borders of regions, whereas 2 coronal reference planes containing anatomical landmarks (posterior tip of the hippocampus and anterior tip of the parieto-occipital sulcus) serve as an anterior/posterior boundary for each region. Importantly, the posterior tip of the hippocampus separated the PTL and the ATL, and therefore contacts located anteriorly to the posterior tip of the hippocampus were labeled in the ATL. The anatomical location of each significant contact was determined in the individual brain according to this anatomical subdivision. The schematic locations of these anatomical structures are shown on a reconstructed cortical surface of the Colin27 brain. AntFG, anterior fusiform gyrus; antMTG/ITG, anterior part of the inferior and middle temporal gyri; antPHG, anterior segment of the parahippocampal gyrus; ATL, anterior temporal lobe; CoS, collateral sulcus; HIP, hippocampus; latFG, lateral part of the fusiform gyrus; medFG, medial part of the fusiform gyrus; MFS, midfusiform sulcus; MTG/ITG, inferior and middle temporal gyri; OCC, occipital lobe; OTS, occipito-temporal sulcus; PHG, parahippocampal gyrus; POS, parieto-occipital sulcus; PTL, posterior temporal lobe; VOTC, ventral occipito-temporal cortex. (TIF) [file pbio.3000659.s007.tif]

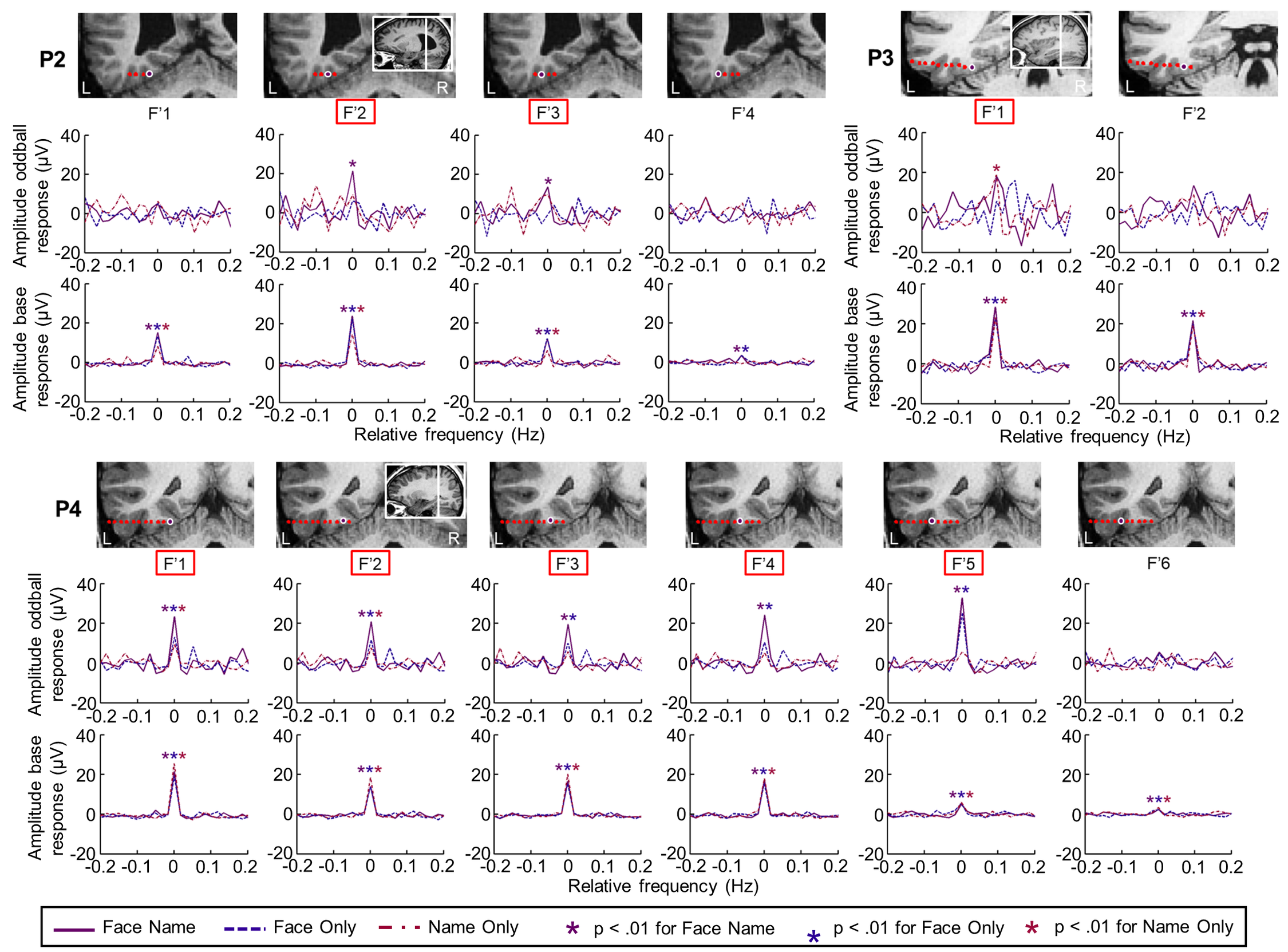

Supplement: S8 Fig — Anatomical location and electrophysiological responses of the 8 significant contacts in the PTL in 3 participants (P2, P3, P4) and of their adjacent contacts. The significant contacts are highlighted in red. Significant base responses were determined in the same way as significant identity-oddball responses by (1) epoching the EEG frequency spectrum into segments centered on the first 6 base harmonics (i.e., 4 Hz, 8 Hz, etc.); (2) summing the amplitude values of these 6 frequency spectrum segments; and (3) transforming it into a Z-score (difference between the amplitude at the base frequency bin and the mean amplitude of the 22 surrounding bins, divided by the standard deviation of amplitudes in the corresponding 22 surrounding bins). Data underlying this figure are deposited on a Dryad repository: https://doi.org/10.5061/dryad.m8t391m. EEG, electroencephalography; SEEG, stereo electroencephalography; PTL, posterior temporal lobe. (TIF) [file pbio.3000659.s008.tif]

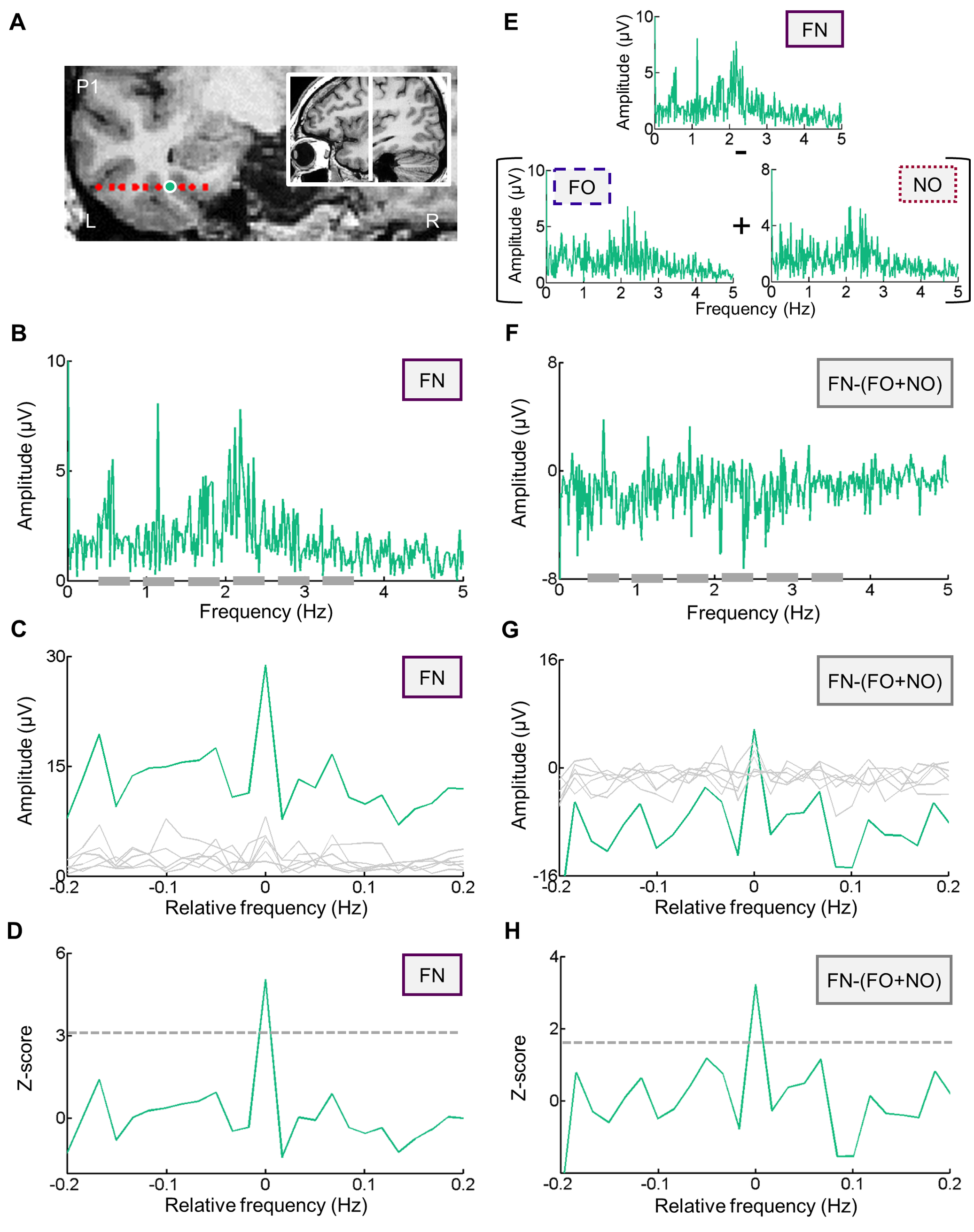

Supplement: S9 Fig — (A) The anatomical location of the contact that is illustrated in this example is shown in both coronal and sagittal views and indicated by a green dot. (B) Intracerebral frequency-domain responses recorded in this individual contact in the Face Name condition. Significant responses were determined by first segmenting the EEG frequency spectrum into 6 segments centered at the identity-oddball frequency and its harmonics. These 6 segments are illustrated by 6 gray bars on the x axis and correspond to the length of each frequency spectrum segment. (C) Pattern of response of the 6 individual frequency segments is shown as gray lines. These segments were then summed, resulting in the green spectrum. The 0 mark represents the identity-change frequency. (D) Z-score transformation of the summed FFT spectrum. Z-score was computed as the difference between the amplitude at the identity-change frequency and the mean amplitude of the 22 surrounding bins, divided by the standard deviation of the 22 surrounding bins. The dashed line indicates the threshold of 3.1 (p < 0.001) that was used in intracerebral EEG to detect significant responses. (E) Computation of the subtraction between the Face Name condition and the sum of the two control conditions: Face Only and Name Only. First, raw FFT spectra of the Face Only and Name Only conditions were summed. This sum was then subtracted from the raw FFT spectrum of the Face Name condition. (F) Intracerebral frequency-domain responses resulting from the subtraction [Face Name − (Face Only + Name Only)]. Significant responses in this subtraction were determined by first segmenting the EEG frequency spectrum into 6 segments centered at the identity-oddball frequency and its harmonics. These 6 segments are illustrated by 6 gray bars on the x axis that correspond to the length of each segment. (G) Pattern of response of the 6 individual segments is shown as gray lines. Because these responses resulted from the subtraction of two conditions ( [file pbio.3000659.s009.tif]
