# Supplementary material for: Neurophysiological evidence for crossmodal (face-name) person-identity representation in the human left ventral temporal cortex
Source: PLoS Biol. 2020 Apr 3;18(4):e3000659. doi: 10.1371/journal.pbio.3000659 (PMC7159237; doi:10.1371/journal.pbio.3000659)
Supplement: Attachment — Submitted filename: Reply-to-reviewers-121219-FINAL.docx [file pbio.3000659.s010.docx]

Reviewer #1:

*My minor comments and suggestions are:*

*Abstract:*

*Change "names of a famous identity" to "names of a single famous identity"*

Reply: We have changed it in the text (p. 2).

*Introduction:

P3 The sentence at the end suggests that verbal information is processed in the right ATL, is this intended?*

Reply: This is a typo, there was an inversion between “left” and “right”. The sentence has been corrected accordingly (p. 4-5).

*P4 I don't know what is meant by "indifferent" neural responses*

Reply: We meant that the neural responses in the bilateral temporal lobes are not specific to the stimulus modality (presentation of a face or a name).

We have changed it in the text (p. 6).

*Methods:

P9 Putin is spelled Poutine, which as far as I know is a Canadian meal of chips and gravy....!*

Reply: We never thought about this ☺

In French, the name is known as “Poutine” indeed (<https://fr.wikipedia.org/wiki/Vladimir_Poutine>) and was thus presented as such to the participants. Since we do not have a figure with the stimulation of that item, we changed it to “Putin” in the text.

*P12 The description of the chunking approach for the visualisation of oddball responses made no sense until I saw the data, it might be helpful to either simplify the text or include a simple diagram to explain this and perhaps other elements of the different oddball response quantification for the non EEG/non FPVS readers.*

Reply: We have simplified it in the text (p. 29-30), and also referred to adequate references where the procedure is fully described (e.g., Retter & Rossion, 2016).

*Figure 1: For the purposes of the figure perhaps keep the font simpler and just use white or black text, it is quite hard to read as it is.*

Reply: We agree with Reviewer#1 that black and white text would be easier to read but we think it is important that the figure reflects the original paradigm (with various fonts and colors) as much as possible for the readers to understand it correctly.

*Results:

P23 The quantification of the presence of significant oddball responses at the individual subject level is not described in enough detail. How was the Z score calculated? While most participants in the Supp Info figure show a clear peak the bottom right participant looks borderline.*

Reply: Z-score computation at the frequency of interest is described in more details in the “Frequency domain analyses” part of Experiment 1. This Z scores calculation applies both to groups and individuals analyses because it relies on the difference between the amplitude at the bin of interest and the average amplitude of the 22 surroundings bins (11 on each side). This difference is divided by the standard deviation of amplitudes in these same 22 surroundings bins, and not on a comparison between subjects.

Some individuals show stronger responses than others indeed as shown on S4 Fig (probably related to interindividual differences), but even if the bottom right participant’s response is of a smaller amplitude than other participants’ responses, the amplitude at the bin of interest representing the response at the identity change frequency is greater than all of the surrounding bins. The z score calculated in this participant is significant at p < .01.

*P24: A frequency plot, similar to Figure 2 in Experiment 1, is definitely needed for Experiment 2.*

Reply: We added a figure in the supplementary material (S5 Fig) showing the FFT spectrum of the responses in the left and right ROI for each condition of Experiment 2.

*P27: Given that there was effectively no oddball response to the Name Only condition, it them seems odd to compare the ROIs of this non-response. I'm not sure this adds much.*

Reply: There is no response in the *Name Only* condition when considering responses across the whole scalp. However, if we consider the response in the ROI, we do get significant responses on the 3^d^ to 6^th^ harmonics in the *Name Only* condition.

To be consistent in our way of presenting the analyses between Experiment 1 and 2, we also changed the text referring to the responses at the frequency of interest in electrodes of the ROI for Experiment 1.

We have modified the text accordingly (p. 10 and 14).

*P29: I think Figure 7 and 6 would be better if they were combined, they are presenting identical types of analyses, just for different ROIs. I also think it would be useful to plot the right ROI. A more general point is does the whole scalp analysis add anything? Being able to show effects at the whole scalp level is great if the effects are strong enough that they can be shown in this way, but if they are not I'm not sure it adds much.*

Reply: We thank Reviewer#1 for this suggestion to combine Figures 6 and 7 and agree that whole scalp analyses can be removed from the paper with no detriment to the results. In the revised version, we have combined figures 6 and 7 into one figure (Fig 5) and removed the whole scalp analyses from the text.

*P29: I don't think the paired t-tests add anything beyond the ROI analysis and this section could be cut with no detriment to the paper. An alternative to this combination of scalp and ROI analyses and separate t-tests would be to use cluster permutation for everything.*

Reply: We agree with Reviewer#1 that paired t-tests add redundant information that is not necessary. This paragraph was removed from the text.

*P30: Did the three patients showing no response show no response at any contact at all?*

Reply: We deliberately restricted our analyses to the left ATL.

Among these 3 patients showing no pure face name response in the left ventral ATL:

- one (patient 7) did not show any response on any contact

- another (patient 4) showed responses on 5 contacts on one electrode of the *posterior* temporal lobe but the responses were not specific to any condition (either significant for all 3 conditions or for the *Face Name* and *Face Only* conditions)

- the other patient (patient 3) showed one contact in the *posterior* occipito-temporal sulcus (posterior temporal lobe) (significant in the *Name Only* condition).

Please see also our response to Reviewer#2 concerning responses in the left posterior temporal lobe that were added to the revised version of the manuscript.

*P31: I'm not clear what is meant by the number of sequences viewed by the participants or the mention of the experiment being repeated?*

Reply: We agree with Reviewer#1 that this part of the text was not clear.

All participants were presented with at least 6 sequences (2 *Face Name*, 2 *Face Only* and 2 *Name Only* sequences), corresponding to one session of the experiment.

In some participants (4 of them), two sessions of the experiment were administered, for a total of 12 sequences (4 *Face Name*, 4 *Face Only* and 4 *Name Only*).

A change was made in the text to clarify this point (p. 18 and 35).

*Discussion:

Without right ATL implants the specificity of the left ATL to this crossmodal representation is unknown but the authors are very clear about this and mention it multiple times.*

Reply: Yes, we agree with the reviewer, our goal was not to claim that the left ATL is the only region showing the crossmodal representation. This was also emphasized by other reviewers and we made sure in the manuscript that we do not claim that our results support a role of the left ATL only.

We sincerely thank the reviewer for their careful reading and evaluation of our manuscript.

Reviewer #2:

*This is a well thought and interesting study; however, the manuscript lacks important considerations of some potential confounds and limitations of the methods. In addition, the scalp EEG results do not provide strong information about responses in the ATLs but they only provide information about occipito-temporal regions. The intracerebral EEG experiment lacks details to determine if results are significant or not. The following issues should be addressed to strengthen this interesting study and make it suitable of publication:*

*Major comments (essential to support current conclusions)*

*- The authors should explain the theory behind frequency-tagging better, cite the first studies using this method (D. Regan, etc), and discuss why they chose to use it. They state in one paragraph in the introduction that they have developed an “original” cross-modal frequency-tagging paradigm. What is original about their method? Other studies have used frequency-tagging to explore cross-modal links (e.g. Colon et al., 2015). What are the advantages over other methods to study cross-modal interactions? E.g. why they do not assess cross-modal interactions measuring ERPs?*

Reply: This is a good point. We have added a paragraph in the introduction to briefly explain the origin and principle of EEG frequency-tagging (p. 6-7). As we mention in the revised version, we used this approach here because of its advantages in terms of sensitivity and mainly, objectivity. That is, we are able to make a very specific prediction for a response in the EEG spectrum to occur exactly at 0.571 Hz. This response can be quantified directly and objectively also (it is much more difficult and subjective with a standard ERP approach because the response could occur at many time points and with different polarities and shapes).

It is true that other studies have used frequency-tagging to explore cross-modal links (e.g. Colon et al., 2015; Saupe et al., 2009; Porcu et al., 2013). However, these studies often had different goals, focusing on how attention to a given modality could affect the response concomitantly recorded (and objectively isolated through frequency-tagging) in another modality. Consequently, the stimulation frequencies are quite different in different modalities (e.g., 7.5 Hz in vision and 20 Hz for tactile stimulation in Porcu et al., 2013), and the studies do not claim that there is crossmodal integration of neural responses.

Here, the different modalities (faces and names) are embedded in the same stimulation sequence.

Note that some studies have used the approach to try to provide objective evidence of integration across modalities (vision and audition) through intermodulation frequency responses (e.g., f1+f2) but apart for a research note without much information (Regan, He & Regan, 1995, in *Experimental Brain Research*) these studies have not been successful (e.g., Giani et al., 2012, *NeuroImage*).

Moreover, our group has extensive experience using the present approach with unimodal “oddball” paradigms (e.g., Liu-Shuang et al., 2014; Rossion et al., 2015; Lochy et al., 2018).

Besides the addition in the introduction section, we also briefly point to the advantages of the approach to identify the crossmodal response with high sensitivity and objectivity in the general discussion section (p. 24).

*- If the objective of the study was to explore the integration of face-name representations, why didn’t the authors design the paradigm with two different frequencies to tag faces and names? This would have given them an indicator of nonlinear interaction, that manifests itself in the form of responses at “intermodulation” frequencies (e.g., f1 ± f2, see Zemon & Ratliff, 1984; Appelbaum et al, 2008; Giani et al., 2012, Boremanse et al., 2013).*

Reply: This is again a very good point, but please see our response above. We are very much aware of this approach (the senior author of the paper has used it in the domain of vision with faces; e.g., Boremanse et al., 2013; 2014; Alp et al., 2016) but there are limitations also of this approach (e.g., many different intermodulation frequencies behaving differently with experimental conditions), which has not been successfully applied to crossmodal interactions (e.g., Giani et al., 2012).

*- What is the spatial location of the authors’ ROIs in a circular view (looking down at the top of the head) topography? Why do the authors consider the source of these signals is in the ATL? They even state that face-name responses were found over posterior sites (second paragraph in Results, Experiment 1). They also do not compare the spatial localization of face-name responses with that of base frequency responses. Thus, how spatially specific are the face-name responses to the ATLs?*

Reply: 2D circular views of the identity-oddball response have been added to Figures 3, 4 and 5 to illustrate the location of our ROI.

A supplementary figure has been added (S6 Fig) to show the spatial localization of base frequency responses and a paragraph has been added in the text to show the statistical comparison of ROI at the base frequency (p. 17). From these analyses, it appears very clear that the response at the base frequency is right-lateralized while the response at the identity-oddball frequency is spread more laterally, on the left and right ROI.

We should also make clear that we do not make any assumption about the source of scalp EEG responses of experiments 1 and 2 being in the ATL just based on these scalp topographies. The ATL was preferentially explored intracerebrally because it is thought to be the source of semantic integration in hub-and-spokes model (as presented in the introduction) and also because most electrode contacts recorded on temporal epileptic patients are located in this left-lateralized region.

We have made this clear in the revised version of the manuscript, and we have also added an analysis of the electrode contacts in the left posterior temporal lobe (p. 18).

*- In Figure 7B, the circular topography shows an area of strong modulation in the amplitude at an anterior right location, maybe in the frontal lobe, or maybe in a fronto-temporal contact. They do not report significant differences here, but it might be because they only report individual paired t-tests in the occipito-temporal regions. Is this amplitude difference significant and in which contact is located?*

**Reply: Following the strong responses in the occipito-temporal regions on both Experiments 1 and 2, our analyses were focused on these regions.**

**On figure 7B, there is indeed a small area responding at the frequency of interest over the right frontal region. However, this response is driven by one electrode only (electrode F6), for which we get a response just above threshold at the frequency of identity change (z = 1.8, p < 0.05). Nearby electrodes do not show any significant response at the frequency of interest (z scores below 1.35), which do not make us confident that this a genuine response. We mention it in the caption of the Fig 5 in the revised version for sake of completeness.**

*- The authors should show that the SNR in all ROIs for the scalp EEG and also in all contacts for the intracranial EEG to determine that the effects are not due to better SNR in some contacts than others.*

Reply: We assess the significance of the responses using a z-score. The z-score is computed as the baseline-subtracted signal (difference between the amplitude at the identity-oddball frequency bin and the mean amplitude of 22 surrounding bins) divided by the standard deviation of amplitudes in the corresponding 22 surrounding bins. The procedure of using the neighbouring frequency bins to the bin of interest as estimation of the EEG noise (rather than say a pre-stimulus baseline) makes sense because the signal is compared to noise in the same frequency range. It has been also validated (e.g., Meigen & Bach, 2000) and used in many studies (e.g., Srinivasan et al., 1999; and all of the studies using the FPVS oddball approach).

In the frequency spectrum, the noise corresponds to the frequency bins adjacent to the frequency bin of interest at the oddball frequency (in this study 22 bins). Hence, we agree with Reviewer#2 that the significance of the responses depends on the amplitude and variance of the surrounding bins recorded on scalp electrodes or intracerebral contacts. This is a fair point.

To take it into account, we extracted the standard deviation of amplitudes in the corresponding 22 bins for each electrode (scalp and SEEG). We compared the noise level between ROI in scalp EEG and found no statistical difference regardless of the conditions. This shows that the noise level was similar across hemispheres in scalp EEG and thus not accountable for the effects that were observed in our study. We added this information in the revised manuscript (p. 11 and 15).

We performed the same analysis for significant and adjacent non-significant contacts in SEEG, i.e. we compared the standard deviation of the noise in all significant contacts (N=15) and in their adjacent non-significant contacts (N=12). Again, there was no statistical difference between these two types of contacts, regardless of the condition. We were not able to compare directly significant and non-significant contacts because non-significant contacts largely outnumbered the significant contact (15 vs. 222). This shows that significant responses on some contacts are not due to local variations of the noise level.

These results are summarized in a new figure (S3 Fig) and in the text (p. 19).

Note that regarding this comment and the next comments below about SEEG, we should mention at this stage that compared to the original version of the paper, we increased the number of significant intracerebral contacts. In addition to the 6 contacts in the ATL reported in the previous version, we added another significant contact in the ATL that we had missed (significant in both *Face Name* and *Name Only* conditions, we apologized for this mistake) and 8 significant contacts located posteriorly to the ATL, in the posterior temporal lobe or PTL (see comment below). We now include a pool of 15 significant contacts in the subsequent analyses.

*Also, for experiment 2, the authors should show if there are differences between hemispheres for the base frequency responses. Otherwise it is unclear whether the differences they see in the Face-name condition and sum of control conditions between hemispheres are due to differences in SNR between hemispheres.*

Reply: There could be genuine differences between hemispheres for the base frequency responses because it reflects a mixture of low- and high-level processes (e.g., the base frequency response at the first harmonic is often right lateralized when only faces are presented at the base rate, e.g., Zimmermann, Yan & Rossion, 2019). This would not imply that there is a SNR difference between hemispheres that affect the oddball response.

That said, following the reviewer’s comment, we added a new figure (S6 Fig) showing the topography and the amplitude quantification of base frequency responses in the 3 conditions of Experiment 2. The scalp topography (right middle occipital) is very similar to what is already shown in Experiment 1.

We also computed a statistical comparison between the left and right ROI in each of the conditions of Experiment 2 at the base frequency. In all conditions, the statistical tests showed a significant difference between left and right ROI, confirming that the scalp topography of the base frequency responses is centred over the right occipito-temporal area.

These statistical analyses were added in the text on page 17.

*- For the intracranial EEG recordings, the methods need to be written and shown in a clearer way.*

Reply: We updated the text in order to explain the intracranial EEG methods in a clearer way (p. 33-38). We also added a new figure (S1 Fig) to explain how we determined the significance of intracerebral contacts.

*-Given that intracranial EEG gives better spatial information than scalp EEG, the authors should show the contacts’ location for each patient in a supplementary figure. Especially important to support the conclusions drawn in this manuscript, the authors should show how anterior these contacts are within the temporal lobes.*

Reply: We added a new supplementary Figure (S8 Fig) showing in the Talairach space the location of all recorded contacts in the 7 patients (in the ATL but also in the posterior temporal lobe, PTL, see the comment below) and the location contacts with significant responses (15 contacts, colored according to their response profile).

*- The authors find that 6 out of the 196 contacts in the pooled intracranial recordings show significant responses at the* *frequency of person identity change. These 6 contacts belong to 4 patients. Where exactly are all contacts located and what do the contacts surrounding these 6 contacts show? What contacts show base frequency responses?*

Reply: As indicated above, we include a pool of 15 significant contacts in the revised version of the paper. We provide 2 new supplementary figures showing the anatomical location of the 15 significant contacts (in both ATL and PTL) in the individual anatomy, their base responses, and the responses of the adjacent contacts of each of these contacts (S7 Fig and S9 Fig).

Identity-oddball frequency responses are only found in the significant contacts and almost no responses are observed at this frequency in the non-significant adjacent contacts. It shows that (1) our statistical threshold is adapted and (2) that there is no or little volume conduction effect. We believe that these observations reinforce our conclusions.

For the base responses, several observations can be made. First, significant base responses are more often found in the PTL than the ATL and showed higher amplitudes in the PTL (compared S7 Fig and S9 Fig). Second, some contacts showed highly significant identity-oddball responses with no base responses (e.g., P1, TB’4, S7 Fig) while others showed significant responses for both identity-oddball and bases responses (e.g., P2, TM’1, S7 Fig). These observations are difficult to interpret without further data, and since they were not relevant for the goal(s) of the paper, they are not discussed.

*- The contact of patient 2 in figure 8 looks very posterior. Why do the authors consider this ATL? They should show clearly in a figure the spatial limits of what they consider ATL.*

Reply: In each individual brain, a coronal reference plane including the posterior tip of the HIP served as the border between PTL and ATL (as in previous work from our group, Jonas et al., 2016, PNAS, Lochy et al., 2018, PNAS). This parcellation is depicted schematically in a new figure (S2 Fig). We considered contacts in the ATL if they were located anteriorly to the posterior tip of the hippocampus. In the Talairach space, this limit corresponds to -40 in the Y axis.

In the revised document, on pages 34 and 37-38, we explicitly define the boundary of the ATL.

*- The authors state that “strikingly” three of 196 contacts show a “pure” face-name association response. I am not sure how striking is this. What is the likelihood of having 3 out of 196 contacts in 7 patients giving this response?*

Reply: The term “strikingly” did not refer to the number of contacts, but to the fact that we were able to find contacts with such a pure “face-name” response. This type of response provides unambiguous evidence of necessary integrated representation of face and name identities. We have rephrased the sentence to make this clearer.

We first considered the ATL because we thought that we had highest chance to find such responses in this region.

We agree with Reviewer#2 we have to clarify what is the likelihood of having 3 pure responses out of 196 ATL contacts in 7 patients.

First, when using patients instead of contacts, 3 out 7 patients showed a pure response when exploring their left ATL, which is quite a high proportion (3/7, 43%).

Second, the proportion significant/recorded contacts in intracranial EEG studies should be interpreted with caution because this proportion highly depends on a series of factors:

-1/ the size of the functional area (the smaller an area, the less likely it is to cross it with electrodes and thus to record a significant response). In this case, we could hypothesize that the functional area integrating faces and names in the ATL is small.

-2/ the number of recorded contacts considered in the proportion depends on the size of the region of interest, which is set arbitrary (usually set before knowing where the significant responses are). For example, our definition of the ATL (see Jonas et al., 2016, PNAS, and Lochy et al., 2018, PNAS) comprises the vATL (antPHG, antCOS, antFG, antOTS), but also lateral part of the ATL (ITG and MTG). Including the lateral part of the ATL, where no face-name responses were recorded but where a high number of contacts were implanted, lowered artificially the proportion of pure responses in the ATL.

This is clearly explained in the revised text on pages 23-24.

*-I do not believe that a strong conclusion can be drawn from this about the role of ATL with this result. There is no control region to compare: maybe 3 out of 196 contacts in pure visual cortex would also give this result?*

Reply: This is a very good point, we agree with Reviewer#2. The 7 patients also had electrodes posteriorly to the ATL (in the left posterior temporal lobe or left PTL), but these electrodes were not included in the original version of the manuscript. In total, we had 41 contacts in the left PTL in 4 patients. We found 8 significant contacts (S9 Fig) in at least one of the 3 conditions (all located in the fusiform gyrus).

However, importantly, we did not find a single ‘pure’ face-name response on these 41 contacts. Two contacts were significant in FN and not in the control conditions NO and FO but the difference [FN– (FO + NO)] was not significant, two were significant in the 3 conditions, three in both FN and NO conditions, and one in the condition NO only. Note that 5 out the 8 significant contacts in the PTL were found in the same patient (see new figure S9 Fig).

**We added the left PTL region in the manuscript and in our analyses.** **We believe that this reinforces our conclusions and we thank the reviewer especially for making this point.**

*Minor comments
- Figure 2 upper panel is not clear. For some frequencies there’s only a blue bar, for some others a red bar, and for others a combination of both. There is no explanation in the legend about this.*

Reply: We clarified the Fig 2 by showing it as two different waves rather than as superimposed bars for each hemisphere. To be consistent with the new figure that was added to show the FFT spectrum in Experiment 2 (S5 Fig), as Reviewer#1 suggested, the results that are presented in Fig 2 now correspond to the response on the pooled electrodes in the left (red line) and in the right (blue line) ROI (3 electrodes on each side).

*- In the third paragraph of the introduction there is a typo:
“On the one hand, verbal and non-verbal person-specific information may be processed separately in the right and left anterior temporal lobe (ATL), respectively”  
In the following sentence they state this correctly: left ATL has been proposed to be part of verbal person-specific information, while right ATL has been proposed to differentially contribute to processing faces.*

Reply: This mistake has been corrected.

*- Abbreviations FSVP, SEEG need explanation where they first appear*

Reply: We apologize for this oversight. The abbreviations are now explained on p. 7 and p. 9.

*- All figures could benefit from a more detailed legend.*

Reply: We have added more details in the legend of most figures.

We sincerely thank the reviewer for their careful reading and evaluation of our manuscript*.*

*Reviewer #3: In normal healthy and epileptic subjects, authors tested the competing hypotheses that verbal and non-verbal person-specific information may be processed separately in the right and left anterior temporal lobe, respectively (Hypothesis 1) or if there is a semantic ‘hub’ in the bilateral anterior temporal lobes, integrating modality-specific representations into a shared amodal/transmodal representation (Hypothesis 2).  In lengthy experiments (whose design and method of analysis are extremely hard to follow) they found "neural" responses that were modality specific and selective to person's identity in the posterior sites (category specific responses are known in this region) but then they also found a modality non-specific semantic "concept" hub in the anterior temporal lobe (also very famously known with single unit recordings in human subjects (as referenced by authors). Sadly, they conclude that this is left lateralized without providing recordings directly
from the right hemisphere. They argue that the lack of right intracerebral recordings was due to scalp EEG findings suggesting unilateral responses--- but lack of scalp findings does not justify lack of recordings. In other words, the paper does not seem to advance any theories and or settle the competition between the two hypotheses.*

Reply: We are sorry that the reviewer found it hard to follow our design and method, and we have tried, following specific recommendations of the other reviewers, to improve their description. We would like to mention that, to our knowledge, a modality non-specific semantic "concept" hub in the anterior temporal neocortex has not been shown with single unit recordings in human subjects: we refer to studies performed in the *Medial* temporal lobe (hippocampus, amygdala) in the manuscript (Quiroga et al., 2005).

The main goal of our paper is to provide original direct evidence for integrated representation of faces and names related to an identity. This goal is achieved with the finding of the oddball responses in EEG (experiment 1), and the exclusion of confounds by comparing to two control conditions (experiment 2).

This finding, in itself, favors the hub-and-spoke model over the alternative hypothesis, *regardless of the lateralization of the effect*. Therefore, our paper *does* provide a theoretical advance and helps settling the competition between the two hypotheses.

In scalp EEG, we mainly find this integration response over the left hemisphere but there is also a trend towards an effect in the same region of the right hemisphere. We make it clear in the paper that our investigation of intracerebral data was not aimed at settling this question because it would require a very large sample of recordings in both hemispheres, which we do not have at this stage. Yet, the SEEG data goes beyond the scalp EEG data by showing ‘pure’ face-name responses. We have made this clear in the manuscript, as also noted by reviewer 1 explicitly (last point).

*Unlike single unit or gamma band recordings in the brain, I have hard time understanding what "neural" signal this 0.5Hz represents. What is so neural about it?*

Reply: A neural response is a response generated directly or indirectly by neurons, and this response can be expressed at multiple scales and in terms of various signals (spikes, postsynaptic potentials). Even a BOLD response in fMRI is typically defined as a neural response. Here we have a direct measure of neural activity (EEG or SEEG), which is expressed as a low frequency change. Thousands of EEG papers refer to neural responses in this context.

*The electrophysiological responses were analyzed at the frequency of oddball presentation 0.571 Hz and its harmonics. Interestingly in Face-Name task this oddball has a more robust structure whereas in the other two tasks the "conceptual" oddball is too randomly and too frequently presented perhaps presenting a confounding effect here. As authors anticipated "conceptual oddball" responses appeared in that signal spectrum exactly at the frequency of person identity change (4Hz/7 = 0.5713 Hz), and one might argue that Exp 2 and 3 did not provide a solid structure for testing the same oddball effect. Only Task 1 did.*

Reply: Experiments 2a and 2b in our paper always included the Experiment 1 in fact. Experiment 2 is a replication of Experiment 1 with the addition of two control conditions. Experiment 2b includes all the conditions of Experiment 2a.

If the reviewer refers to the three experimental conditions, the reasoning is the following:

A population of neurons sensitive to face identity only (= “blind” to names) should generate the same level of oddball activity in the conditions “Face Name” and “Face Only” depicted on Figure 1. On average, 50% of the time, every 1/7 stimuli, a different face than D. Trump is shown. For both conditions.

The same reasoning holds for the Name only condition relative to the “Face Name” condition.

If there is no population of neurons sensitive to both faces and names, the two independent populations of neurons will discharge in the “Face name” condition. Hence, the sum of “Face Only” and “Name Only” should be the same as the response obtained in the “Face Name” condition.

However, if there is also a population of neurons that is sensitive to both faces and names, it should generate an additional response in the “Face Name” condition.

*Also I have a problem with including 6 different colors for names and yet no specific color change for faces. Also face images subtended about 6.78° x 5.47° of visual angle, but the name condition subtended 13.65° x 1.62° of visual angle.*

Reply: This is a fair point but the goal of study is not about comparing the magnitude of the response to faces and words. Face images of the same person differ in many more aspects than words (background luminance and color, viewpoint, size, lighting, etc.). These variations are used to ensure that the detection of the change of identity is not due to low-level characteristics of the faces (as in studies with faces only relying on this type of design, see Zimmermann, Yan & Rossion, 2019).

Variations in size, font and color for words are introduced for the same purpose. One cannot make words vary the same way as faces, and it would be incorrect to try to present faces and words at the same size.

In the revised version, we have justified the use of variable stimuli (p. 25).

Even though the reviewer does not seem convinced by our study, we have tried our best to reply and take into account the comments in the revised version. We thank the reviewer for their careful reading and evaluation of our manuscript.

*Reviewer #4:

Specific points:

Abstract
It might be useful to note that semantic information (about people and any other concept) is more than just the association of faces and names. A key central aspect of semantic concepts is the ability to generalise knowledge across all modalities and over time. Thus there are many forms of knowledge, including names, that are linked to each person.*

Reply: We agree that the association of faces and names is only a small aspect of semantics. We have made this explicit in the revised introduction of the manuscript (p. 6):

“*Regarding the specific association of familiar faces and names, which is only a subcomponent of person semantics …”*

*Like the main text, the abstract discusses left vATL functions with an implicit message that the results are different for other regions. Whilst the study can make some excellent observations about the left vATL, because the right vATL was not sampled then little can be said about it. Note – the broader semantics literature does have a consideration of left and right vATL functions and how they might work together.*

Reply: We agree that we cannot make conclusions about the right ventral ATL and have been clear about this throughout the paper. In the revised version of the paper, we have emphasized much more the contribution of our study in terms of finding evidence for an integrated neural representation of faces and names at the level of a specific person identity, irrespective of the hemispheric lateralization issue (introduction: p. 6; discussion: p. 20 and 22-23).

*Introduction
p.4: I think that it is important to note that the difference between left and right SD patients is graded with a _relative_ difference between words and faces. It is never an absolute difference and not a classical double dissociation. The same graded differences are observed in TLE resection patients and in functional neuroimaging (see various papers by Rice et al. 2015; 2017)*

Reply: We agree with the Reviewer’s view and changed the text to emphasize this graded and relative aspects explicitly (p. 5):

“*Consistent with these studies, group studies of SD patients have shown that the recognition of famous names is relatively more affected by a left anterior temporal atrophy, while famous face recognition deficits are relatively more affected by a right anterior temporal atrophy (11,33,34). Neuropsychological single-case studies of patients with anterior temporal atrophy (35–38) or focal anterior temporal damage (39) have also found evidence for graded hemispheric dissociations within person-related recognition abilities, i.e. relative differential impairments in accessing person-related information from faces or names depending on the hemispheric side of the lesion.”*

*p.4-5: important to note that the hub-and-spoke model provides a much broader framework for semantic memory in general including knowledge about people. The same model shows that activating semantic information to the specific level is demanding and thus is more vulnerable to damage (see Rogers et al 2004; Rogers & Patterson 2007; Rogers et al 2015)*

**Reply: We have tried to make it clear in our introduction that our study is not aimed at questioning the hub-and-spoke model, but to test one specific aspect of this model, which is the face-name integration at the level of a given identity. We also aimed at quantifying the relative contribution of modality-specific responses and the amodal semantic representation.**

*p.4-5: In terms of a lack of evidence about integrated representations for verbal and visual forms of people: (a) if we assume that this is part of general semantics then there is converging evidence for multimodal semantic representations in semantic dementia, fMRI, TMS, computational models, etc. (for an overview, see Lambon Ralph et al NRN 2017) – with, for example, Visser et al (2011) showing that the same vATL region as observed in the SEEG in this study is activated by pictures, sounds and spoken names; (b) SD patients show especially poor performance for semantic knowledge probed at the specific level including people and show strong item associations when knowledge for a concept is probed using words or pictures (indicating degradation of a shared representation); (c) at least one fMRI study has probed people knowledge with pictures and words (Rice et al 2017 Proc Roy Soc) which found bilateral vATL regions that responded to both the spoken names and pictures of familiar people, landmarks and animals.*

Reply: The reviewer is correct and our goal is not to test/question the spoke-and-hub model in general in the paper. Our goal is to test evidence for an integrated representation of faces and names for a specific identity, and then discuss whether the findings are compatible with a spoke-and-hub model (which predicts such a representation) or a model in which the representation of specific identities is kept separated.

Therefore, we think that the evidence provided in our study is both more specific (i.e., it concerns only the identity of people) and direct. In semantic dementia, one cannot exclude that distinct neural representations of identity for faces and names are degraded. The fMRI study of Rice et al. (Phil Trans R Soc, 2018) nicely shows that bilateral vATL regions respond to both names and face pictures, but it is unclear if there is integration at the level of a given identity, as we discuss in the introduction of our paper. In addition, our study suggests that in addition to modality-specific representations of familiar identities, there is an integrated representation *beyond* that. Also, we can quantify the contribution of these responses with the EEG experiment 2.

In the revised version, we have specified these points in the text (p. 6).

*Methods:
Could you say something about how the baseline frequency was determined. I believe in previous face-only FPVS experiments, a higher frequency was used. I assume that finding the correct frequency is critical.*

Reply: The baseline frequency determines the stimulus duration basically, which is of about 250ms here. The reviewer is correct that we usually stimulate faster, e.g. 6 Hz, for visual face discrimination. Different functions are associated with optimal “oddball” responses at different frequencies but there is no evidence that the nature of the response changes with frequency rate, only its amplitude (Retter et al., 2019, https://www.biorxiv.org/content/10.1101/658625v1). Here we assumed that the activation of an integrated semantic representation would slightly require more time, and therefore we were conservative by using a 4 Hz frequency rate.

*Discussion
If I have understood correctly then only the left vATL was sampled in the SEEG? And scalp EEG cannot be used for localising responses in these anterior regions. I think it is important to make it clear, therefore, that we cannot consider what the right vATL might be doing from these data. Parallel work from semantic dementia, fMRI, rTMS and subdural grids suggests a bilateral division of function perhaps with some graded differences that align with connectivity variations (see Rice et al., for reviews and meta-analyses). And combined rTMS-fMRI suggests that the division of labour across left and right can change in line with demand (see Binney et al 2015; Jung et al 2016). Accordingly, the current study gives us interesting insights about the left vATL but much less about other regions.*

Reply: We agree with the reviewer that no conclusion can be drawn about the role of the right ventral ATL in face-name integration from our intracerebral data and our aim was not to say that the left ventral ATL is the only region to contribute to face-name integration.

We are aware that additional intracerebral recordings in the right ventral ATL are necessary to be able to disentangle the respective role of the left and right ventral ATL in this integrative process.

We have emphasized this point in the manuscript (p. 23):

“Nonetheless, it is important to point out that our findings shedding light on the left ventral ATL do not preclude a similar involvement of its right hemispheric counterpart, which was not explored in SEEG in the present study. We therefore emphasize again the need for additional intracerebral recordings in large populations of participants (e.g. 77,78) in the bilateral ventral ATL in order to clarify its role in crossmodal person-related processing. ”

*In this context on p.34, I do not understand the statement “Such a unilateral face-name locus was unexpected considering that proponents of the unitary “hub” theory postulate that both ATL contribute to and are activated during multimodal semantic processing”. As noted by the authors in the previous sentence the right vATL was not probed with SEEG and thus this statement rests on the scalp distributions from EEG.*

*Past work has already show that (a) laterality differences seems to be much stronger in posterior than anterior temporal regions (see Rice et al. reviews and meta-analysis, and Hoffman et al Cortex 2018); (b) the anterior temporal areas are both engaged by verbal and nonverbal stimuli (e.g., Visser et al) and (c) that these bilateral involvements are asymmetric for any tasks that require speech production of written word reading (see Rice et al 2015 meta-analysis). There are various computational models of the hub-and-spoke model that show how these asymmetric bilateral involvements can follow from differences in functional/structural connectivity (e.g., Lambon Ralph et al 2001; Shapiro et al 2013).

p.34-35: please see comments above for the Introduction about at least one previous study that has found a common (bilateral) vATL involvement in both faces and names at the specific level (Rice et al Proc Roy Society).*

**Reply: We agree that this statement relies on scalp EEG data alone and cannot apply to SEEG data because of the lack of recordings in the right vATL.** **We have made this clear in the revised version of the manuscript (p. 21 and 23). We cannot, of course, claim that the left-lateralized effect found on the scalp comes from anterior temporal lobe regions rather than posterior regions (and we do not think that this could be clarified with source localization procedures, because the source localization problem is undetermined). However, in SEEG, we failed to find any electrode contact with a larger response for the FN condition than the sum of the two other conditions in the posterior regions (PTL). Moreover, even if our effect originates from posterior regions, there would be no reason to expect a left-lateralized effect in a paradigm that does not require speech production of written word reading but only an orthogonal task, and involves as many verbal and pictorial stimuli.**

**That said, even if the nonlinear EEG response is left lateralized visually, we did not find a significant interaction between the *ROI* and the *Conditions* in our main analysis, so that we made it clear in the discussion that our findings are not at all inconsistent with the hub-and-spoke theory.**

We sincerely thank the reviewer for their careful reading and evaluation of our manuscript.
