# Supplementary material for: Neurophysiological evidence for crossmodal (face-name) person-identity representation in the human left ventral temporal cortex
Source: PLoS Biol. 2020 Apr 3;18(4):e3000659. doi: 10.1371/journal.pbio.3000659 (PMC7159237; doi:10.1371/journal.pbio.3000659)
Supplement: Attachment — Submitted filename: Reply-to-reviewers_2_FINAL.docx [file pbio.3000659.s011.docx]

Reviewer #1, George Stothart:

*My comments were relatively minor and mostly suggested removing redundant analyses, which the authors have done to my satisfaction.*

Reply: We thank again the reviewer for his very useful comments on our manuscript.

Reviewer #2:

*Although I find this study interesting, and the authors addressed each of my points, I do not think that any strong conclusions can be made about the role of the anterior temporal lobes in cross-modal processing. For this manuscript to be published, the conclusions drawn about the role of the left ATL in the integration of face and name information, and the last sentence of the abstract must be changed in order to reflect the limitations that the study has.*

Reply: The role of the ATL in cross-modal processing is well-known from other studies in the literature, and again this is why this region was targeted here. According to the Reviewer’s comment, we have nevertheless changed the last sentence of the abstract to be less clear cut about the role of the left ATL in the integration of famous faces and names (p. 2, replacing “direct evidence” and “pointing to a key role” by “highlighting the contribution of”).

*Scalp EEG results point towards a general occipito-temporal role in the integration of verbal and non-verbal person identity-specific information. After reading the responses to my and other reviewers' comments, and all the changes the authors made, I agree that this is a strong result. However, I still do not see how the intracranial EEG support these results. First, recordings are restricted in each patient only to the left hemisphere. They do not find any pure face-name response on the PTL contacts (out of 41) but they find 3 contacts in the ATL (out of 196) that show a pure face-name response. Again, I do not see how this can be statistically significant. What is the likelihood of having 3 significant contacts out of 196 vs. 0 out of 41 contacts by chance? In addition, how having 3 out of 7 patients showing this result is a high proportion? I believe the way intracranial EEG was applied as a method* *is not suitable to respond the questions the authors posited in the introduction.*

Reply: Although we respectfully disagree that SEEG is not suitable to respond to the questions asked in the introduction, we understand the Reviewer’s point of view and therefore we: (1) clearly mentioned that we recorded only few ‘pure’ face-name responses, both in the abstract and in the discussion section and (2) we reinforced the paragraph showing that a low proportion of significant responses in SEEG should be interpreted with caution and highly depends on the size of the region of interest, which is set arbitrary (i.e., if we restrict our region of interest to the left anterior fusiform, our proportion increases to 3 pure face-name response out of 26 recorded contacts).

*Finally, the authors should be careful when talking about neural populations, since they do not test this directly. Even if an area acts as a crossmodal hub, unless the activity of single neurons is analyzed, nothing can be concluded about the neural populations per se. Even with the intracranial EEG, the frequency tagging could reflect the existence of two different neural populations within the vicinity of the contact, one responding to faces and one to names. Unless single units are recorded and analyzed no conclusion can be made about the existence of a crossmodal population of neurons (this should also be modified in the abstract as well as in the main body of the manuscript).*

Reply: According to the Reviewer’s comment, we added a sentence in the discussion to address this issue (p. 23). We also removed the term “neural populations” when it was not necessary. We referred instead to “neural activity” throughout.

Reviewer #3:

*I thank the authors for their replies.*

Reply: We thank again the reviewer for their helpful comments.
